# Phase separation of p85β modulates hepatocellular carcinoma progression through POLR1A

**DOI:** 10.7150/ijbs.130598

**Published:** 2026-05-18

**Authors:** Yifan Zhang, Dong Zhang, Yi Duan, Gaoping Cui, Yanhua Zhang, Baoyu He, Meilian Yao, Xiangyu Li, Chengkun Chen, Zhe Li, Shiyi Yang, Jiahao Zheng, Jun Gu, Dongmei Zhang, Yongzhong Liu, Zhang Lin, Hualian Hang, Yujun Hao

**Affiliations:** 1 State Key Laboratory of Systems Medicine for Cancer, Shanghai Cancer Institute, Renji Hospital, School of Medicine, Shanghai Jiao Tong University, Shanghai, 200032, China.; 2 Department of Medical Oncology, Fudan University Shanghai Cancer Center; Department of Oncology, Shanghai Medical College, Fudan University, Shanghai 200032, China.; 3 Department of Laboratory Medicine, Affiliated Hospital of Jining Medical University, Jining Medical University, Jining, Shandong, 272029, China.; 4 Department of Liver Surgery, Renji Hospital, School of Medicine, Shanghai Jiao Tong University, Shanghai, 200127, China.; 5 Shanghai Cancer Institute, School of Biomedical Engineering, Shanghai Jiao Tong University, Shanghai, 200030, China.; 6 Medical Research Center, Affiliated Hospital 2 of Nantong University, Nantong, Jiangsu, 226001, China.

**Keywords:** p110-free p85β, liquid-liquid phase separation, hepatocellular carcinoma, POLR1A, circular RNA

## Abstract

PI3K complex consists of catalytic subunit p110s and regulatory subunit p85s. Emerging evidence indicates that p110-free p85 subunits play pivotal roles in diverse biological processes, including cancer progression. In this study, we demonstrate the underlying mechanism of p110-free p85β in hepatocellular carcinoma (HCC) development. PIK3R2/p85β is upregulated in HCC and correlates with poor patient survival. Nuclear p85β, but not its cytoplasmic counterpart, exhibits oncogenic activity. In the nucleus of HCC cells, p85β undergoes liquid-liquid phase separation (LLPS) and specifically accumulates in the fibrillar centers of nucleoli, where it drives HCC progression. Within the nucleolar compartment, p85β interacts with and stabilizes POLR1A, the catalytic core subunit of RNA polymerase I, thereby enhancing rRNA biosynthesis and maintaining HCC stemness. Furthermore, we develop an engineered circular RNA that encodes a peptide containing p110α ABD domain, which effectively suppresses HCC tumor growth by simultaneously disrupting p85β/POLR1A condensates and inhibiting PI3K/AKT signaling pathway, offering a novel RNA-based therapeutic strategy against HCC.

## Introduction

The phosphoinositide-3 kinase (PI3K)/AKT signaling pathway is one of most frequently dysregulated pathways in human cancers, including hepatocellular carcinoma (HCC) - the predominant form of primary liver cancer and a leading cause of global cancer-related morbidity and mortality 1-3. PI3K complex comprises regulatory subunits (p85α and p85β) and catalytic subunits (p110α, p110β, p110δ and p110γ). Upon stimulation by growth factors or insulin, PI3K complex are recruited on cell membrane to generate phosphatidylinositol 3,4,5-trisphosphate (PIP3) from phosphatidylinositol 4,5-trisphosphate (PIP2) 4. As a second messenger, PIP3 binds and activates multiple downstream effectors such as AKT and PDK1 through their pleckstrin homology (PH) domains, thereby initiating a cascade of signaling events that drive tumorigenesis 4.

Regulatory subunit p85s normally stabilize p110s and regulate their lipid kinase activity, thereby being involved in cell growth, migration and apoptosis. However, emerging evidence reveals that p85s can also execute diverse biological functions independent of catalytic activity of p110s. For instance, p85α directly binds to PTEN, enhancing its lipid phosphatase activity 5. Additionally, p85α can activate CDC42 to mediate cytoskeletal reorganization by PI3K-independent mechanisms 6. Both p85α and p85β facilitate the nuclear translocation of XBP1 (X-box binding protein 1), thereby regulating endoplasmic reticulum stress responses 7-9. Furthermore, p85α and p85β mediate the interaction between BRD7 (bromodomain-containing protein 7) and XBP1 to maintain cellular glucose homeostasis 10. Moreover, p85β promotes tumorigenesis through interacting with histone methyltransferases EZH1/EZH2 11, 12. Notably, Tsolakos *et al*. have discovered that more than 30% of p85s are p110-free forms in mouse liver 13, suggesting that p110-free p85s might play important role in liver physiology including HCC progression.

Eukaryotic cells contain numerous membraneless organelles, including nucleoli and stress granules, which facilitating spatiotemporal regulation of cellular functions and diseases 14. Liquid-liquid phase separation (LLPS) has emerged as a fundamental mechanism driving the formation and function of membraneless organelles 15. Growing evidence highlights the crucial roles of membraneless organelles in tumorigenesis 16. For instance, genetic ablation of long noncoding RNA NEAT1 disrupts p53-induced nuclear body paraspeckles, thereby preventing skin tumor development in mice 17. Similarly, depletion of G3BP1, a core component of stress granule, significantly influences the expression of epithelial-mesenchymal transition (EMT) markers including Cadherin, Vimentin, Snail and Slug, suggesting the role of stress granules in tumor metastasis 18. Notably, increased nucleolar number and size are hallmarks of certain cancers 19, and elevated expression of POLR1A, the largest subunit of the RNA polymerase I in nucleolus, has been characterized as a key feature in the stem cell hierarchy driving colorectal cancer progression 20. Recent studies have further elucidated the oncogenic mechanisms of various cancer-associated proteins, including SHP2 mutants, nuclear YAP, and EML4-ALK fusion proteins, through their phase separation properties 21-24. However, whether PI3K isoforms exert their oncogenic functions through LLPS remains unexplored.

In this study, we uncover a PI3K-independent, phase separation-mediated oncogenic mechanism of p85β in HCC. We demonstrate that p85β undergoes LLPS to form biomolecular condensates, which specifically is localized in fibrillar centers of nucleolus in HCC cells. In the nucleolus, p85β interacts with and stabilizes POLR1A, thereby enhancing rRNA biosynthesis to drive HCC progression. Importantly, we developed an innovative therapeutic strategy using engineered circular RNA to deliver a p85β-binding peptide. This engineered circRNA shows potent anti-tumor efficacy through its dual functions to target both p85β/POLR1A condensates and PI3K/AKT signaling pathway.

## Results

### PIK3R2/p85β promotes hepatocellular carcinoma tumorigenesis

**Figure 1:**
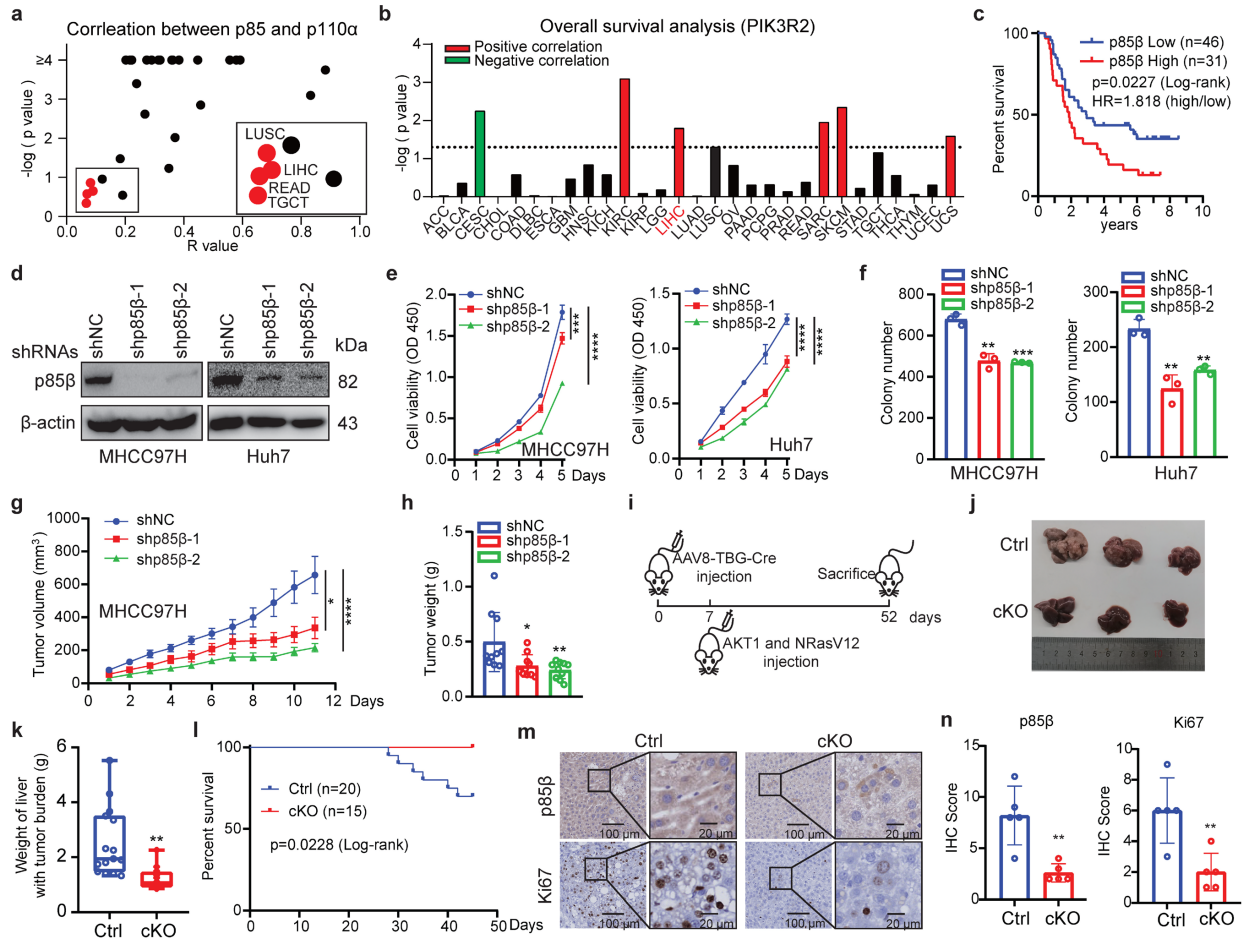
** p85β promotes HCC progression. a** The correlation analysis of protein levels between p110α and p85 by RPPA database. **b** The correlation between PIK3R2/p85β expression and overall survival of patients in various cancer types with TCGA database. **c** High expression of p85β is correlated with worse overall survival of HCC patients by tissue microarray. **d-f** p85β knockdown inhibits the growth of HCC cells. Control cells (shNC) and p85β knockdown cells (shp85β) were assayed for knockdown efficiency (**d**), proliferation rates by cck-8 (n=5) (**e**) and colony formation ability (n=3) (**f**). **g-h** p85β knockdown inhibits the growth of subcutaneous xenograft tumors. The growth curves **(g)** and the final tumor weights **(h)** of MHCC97H-generated subcutaneous xenograft tumors were plotted (n=10). **i-n** The tumorigenesis of spontaneous hepatocellular carcinoma was impaired by p85β knockout. Wild-type (Ctrl) and *Pik3r2* conditional knockout (cKO) mice were injected with AAV8-TBG-Cre virus followed by injection of myr-AKT1 and NRasV12 plasmids **(i)**. Representative mice livers **(j)**, liver weights with tumor burden (Ctrl=15, cKO=12) **(k)**, and overall survival curves (Ctrl=20, cKO=15) **(l)** of Ctrl and cKO mice were plotted. Representative IHC staining of p85β and Ki67 in liver tumors from Ctrl and cKO mice were shown **(m).** Quantification of IHC staining of p85β and Ki67**(n)**. *p < 0.05, **p < 0.01, ***p < 0.001, ****p < 0.0001.

To investigate the functional significance of p110-free p85s in hepatocellular carcinoma (HCC), we first analyzed TCGA reverse-phase protein array (RPPA) data. While p85s and p110α protein levels showed strong correlation across most cancer types (Fig. 1a), consistent with their canonical interaction in PI3K complexes, four tumor types including HCC (LIHC), lung squamous cell carcinoma (LUSC), rectal adenocarcinoma (READ), and testicular germ cell tumors (TGCT) exhibited discordant expression patterns (Fig. 1a). This dissociation suggests potential PI3K-independent roles of p85s, particularly in HCC tumorigenesis. Notably, survival analysis revealed that PIK3R2/p85β, but not PIK3R1/p85α, was highly expressed and correlated with worse overall survival exclusively in LIHC patients among these four cancer types (Fig. 1b, S1a and S1b). The elevated expression levels of PIK3R2/p85β or its correlation with worse overall survival of HCC patients in both TCGA and ICGC datasets (Fig. S1c and S1d). The correlation between p85β expression and overall survival of HCC patients was further confirmed by Renji hospital cohort (Fig. 1c), implying p85β as a critical player in HCC tumorigenesis. Moreover, as shown in Fig. S1e-f, we analyzed the expression of PI3K catalytic subunits in HCC using TCGA and ICGC datasets. The results showed that PIK3CA and PIK3CB, but not PIK3CD and PIK3CG were highly expressed in HCC. But, the expression of PI3K catalytic subunits were not well-correlated overall survival of patients. Those results showed that p85β may play a key role in HCC.

We further confirmed the oncogenic function of p85β in HCC. We examined the endogenous expression levels of p85β in HCC cell lines (MHCC97H, Huh7, SNU449, Hepa 1-6, HCC-LY10 and Hep 3B) (Fig. S3a). Firstly, p85β knockdown significantly inhibited the proliferation and colony formation of HCC cells (Fig. 1d-1f). Secondly, knockdown of p85β markedly reduced the growth rates and final weights of xenograft subcutaneous tumors generated by MHCC97H cells (Fig. 1g, 1h, and S1g). Finally, we established a *Pik3r2* conditional knock-out mouse strain to validate the oncogenic function of p85β with spontaneous HCC model (Fig. S2a). Both wild-type (Ctrl) and *Pik3r2* conditional knockout (cKO) mice were administrated with AAV8-TBG-Cre virus followed by tail vein injection of activated NRasV12 (N-Ras G12V) and AKT1 (myr-AKT1) to induce HCC (Fig. 1i). Comparative analysis revealed that *Pik3r2* cKO mice exhibited less liver tumor burden and liver weights (Fig. 1j and 1k), resulting in prolonged survival compared to control mice (Fig. 1l). Immunohistochemistry confirmed the efficiency of p85β depletion and reduced proliferative capacity in cKO mice liver tumors, as evidenced by p85β or Ki67 staining respectively (Fig. 1m and 1n). Those data suggest that p85β functions as an oncogene in HCC progression.

### p85β nuclear translocation is essential for its oncogenic function

**Figure 2:**
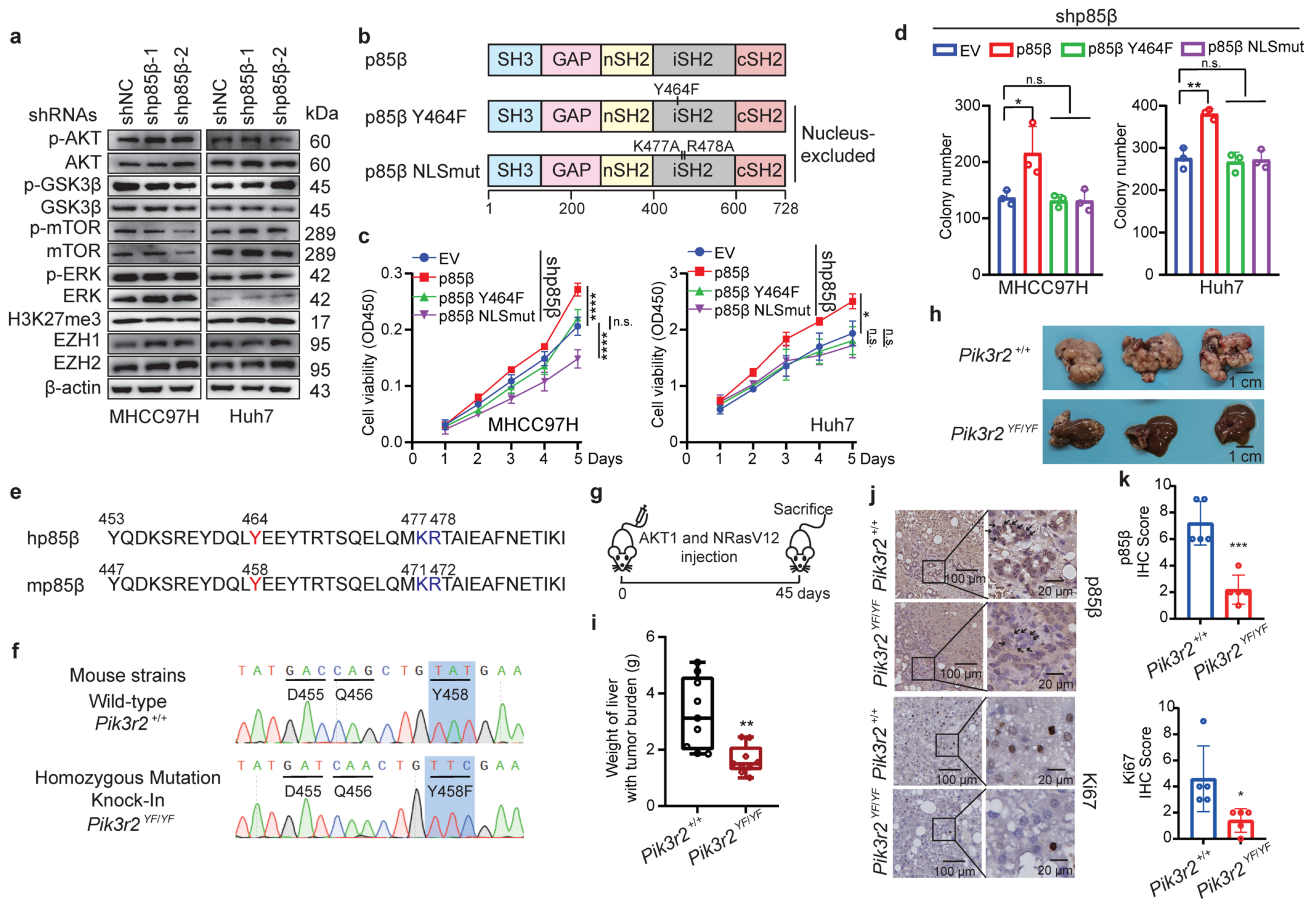
** p85β nuclear translocation is critical for its oncogenic function in HCC. a** p85β knockdown had no impact on AKT signaling and H3K27me3 in HCC cells. **b-d** Nucleus-excluded p85β cannot rescue the growth defect caused by p85β knockdown. The schematic of p85β nucleus-excluded mutant proteins (**b**). Wild-type but not nucleus-excluded p85β rescued the proliferation (n=5)** (c)** and colony formation ability (n=3)** (d)** of p85β knockdown HCC cells. **e-j** Blocking nuclear translocation of p85β impaired spontaneous hepatocellular carcinoma tumorigenesis. The core nuclear localization signal sequence (blue) and tyrosine phosphorylation sites (red) determining p85β nuclear translocation are completely conserved between human (hp85β) and mouse (mp85β) **(e)**. The DNA sequences of wild-type (*Pik3r2^+/+^*) and homozygous mutation knock-in (*Pik3r2^YF/YF^*) mice surrounding mPik3r2 Y458 were presented **(f)**. Spontaneous hepatocellular carcinoma was induced by injection of myr-AKT1 and NRasV12 plasmids **(g)**. Representative liver tumor pictures **(h)** and liver weights with tumor burden **(i)** (*Pi3kr2^+/+^* =9, *Pi3kr2^YF/YF^* =9) were shown**.** IHC staining for p85β and Ki67 in liver tumors were presented **(j)**. Quantification of IHC staining of p85β and Ki67 (n=5)** (k)**. Black arrows indicated that *Pi3kr2^Y458F^* mutation blocked the nuclear translocation of p85β. n.s. not significant; *p < 0.05, **p < 0.01, ***p < 0.001, ****p < 0.0001.

While p85β is known to modulate classic AKT signaling in the cytoplasm or histone methyltransferase EZH1/2 in the nucleus to promote tumorigenesis 11, 12, 25-27. However, knockdown of p85β in HCC cells did not reduce the phosphorylation levels of AKT, mTOR, GSK3β, and ERK, or the protein levels of EZH1 and EZH2 (Fig. 2a), suggesting that p85β promotes HCC growth through an unknown mechanism. To further explore that p85β performs oncogenic function in cytoplasm or nucleus of hepatocellular carcinoma cells, various p85β constructs were evaluated for their ability to rescue the growth defect caused by p85β knockdown (Fig. 2b). Notably, only overexpression of wild-type p85β, but not nucleus-excluded p85β mutants (p85β Y464F or p85β NLSmut) 12, restored the proliferation and colony formation of p85β knockdown cells (Fig. 2c-2d). This highlights the critical role of nuclear p85β in HCC tumorigenesis.

Nuclear translocation of human p85β (hp85β) depends on phosphorylation at Y464 and a nuclear localization signal (NLS) sequence near Y464^12^. Mouse p85β (mp85β) shares an identical tyrosine residue at Y458 and NLS sequences as hp85β (Fig. 2e). To further validate the oncogenic function of nuclear p85β *in vivo*, a transgenic mouse strain with an isogenic point mutation *Pik3r2^Y458F^* was generated (Fig. 2f and S2b). After injection of NRasV12 and AKT1, homozygous mutation knock-in *Pi3kr2^YF/YF^* mice exhibited less liver tumor burden and liver weights compared with that of wild-type (*Pi3kr2^+/+^*) mice (Fig. 2g-2i). Importantly, *Pi3kr2^YF/YF^* mice showed similar mRNA and protein levels of Pik3r2/p85β as *Pi3kr2^+/+^* mice (Fig. S2c and S2d), whereas exhibited less nuclear translocation of p85β in mouse liver tumors (Fig. 2j and 2k). These data suggest that p85β promote HCC tumorigenesis through its p110-free form in the nucleus.

### p85β forms condensates in nucleus by liquid-liquid phase separation

**Figure 3:**
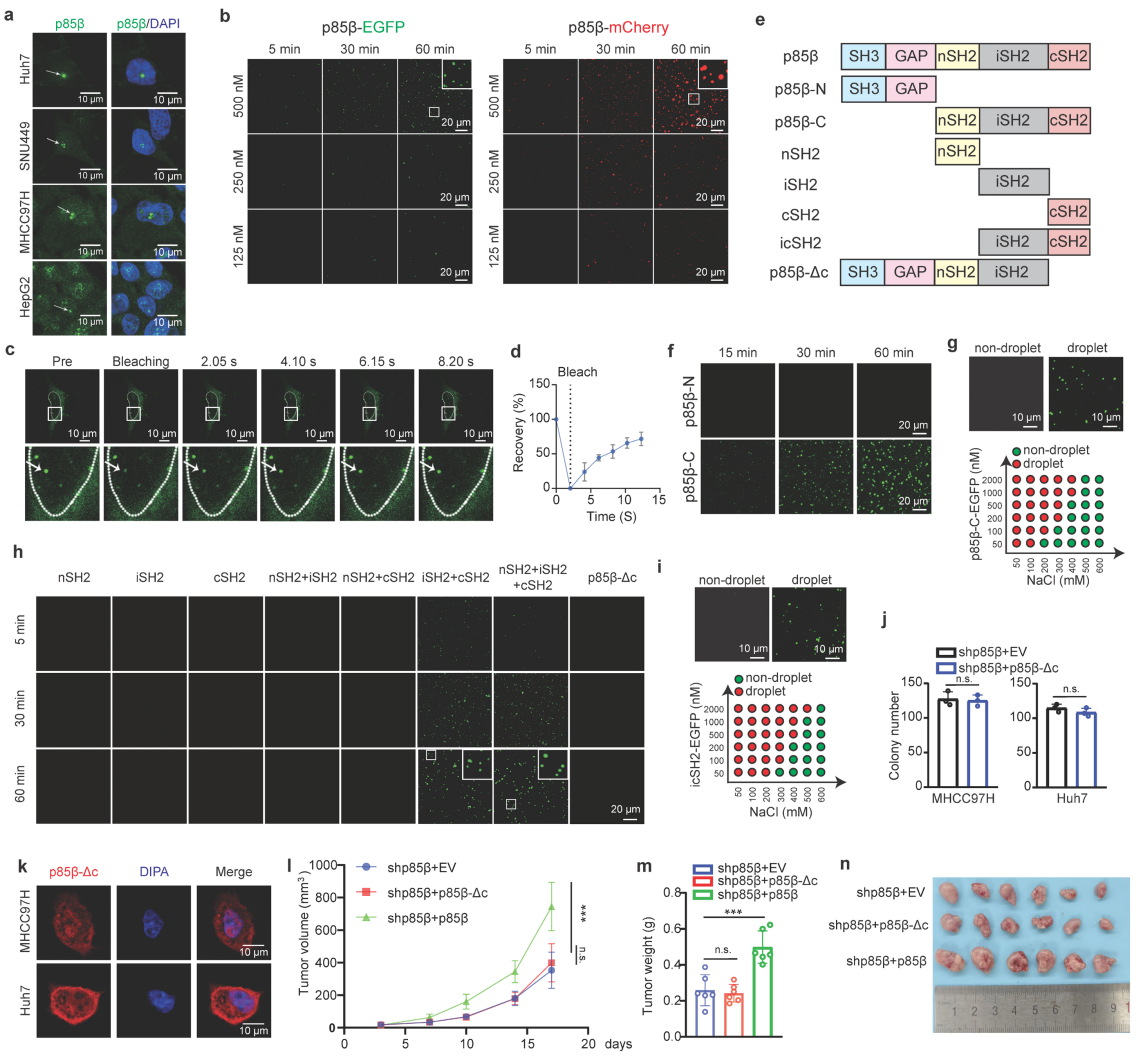
** Phase separation of p85β in the nucleus promotes HCC progression. a** p85β forms condensates in the nucleus in HCC cells. **b-d** p85β undergoes LLPS. Recombinant p85β-EGFP or p85β-mCherry fusion proteins forms condensates *in vitro* **(b)**. p85β-EGFP formed condensates in the nucleus in Huh7 cells **(c)** and the condensates were quickly recovered after photobleaching (FRAP assay) (n=5) **(d)**. **e-i** iSH2 and cSH2 domains are important for phase separation of p85β. The schematic of p85β truncated proteins (**e**). C-terminal of p85β including nSH2, iSH2 and cSH2 underwent LLPS (**f, g**). Co-existence of iSH2 and cSH2 exhibited phase separation and p85β-Δc failed to form condensates (**h, i**). Deletion of cSH2 to disrupt LLPS in p85β fails to rescue the growth defect caused by p85β knockdown. p85β-Δc failed to form condensates in the nucleus in HCC cells **(k)**. p85β-Δc overexpression failed to promote colony formation (n=3) (**j**) of p85β knockdown cells. **l-n** overexpression of p85β-Δc failed to restore the growth defects caused by p85β knockdown of xenograft tumors The growth curves **(l)** and the final tumor weights **(m)** of MHCC97H-generated subcutaneous xenograft tumors were plotted (n=6). n.s. not significant, ***p < 0.001.

Intriguingly, p85β tended to form liquid-like droplets in the nucleus of HCC cells (Fig. 3a). To confirm this observation, we generated EGFP- or mCherry-fused p85β constructs and purified these recombinant proteins for *in vitro* analysis (Fig. S4a). Both p85β-EGFP and p85β-mCherry formed liquid droplets *in vitro* (Fig. 3b). Similarly, p85β-EGFP droplets were observed in the nucleus of HCC cells (Fig. 3c), and displayed rapid recovery ability after photobleaching (FRAP) assays (Fig. 3c and 3d). These findings indicate that p85β undergoes liquid-liquid phase separation both *in vitro* and *in vivo*.

To determine the domains responsible for driving LLPS in p85β, we generated a series of EGFP-fused p85β truncation mutants and evaluated their phase separation capabilities (Fig. 3e and Fig. S4a). First of all, p85β C-terminal including nSH2, iSH2 and cSH2 domains, but not N-terminal, exhibited phase separation (Fig. 3f and 3g). Surprisingly, none of individual SH2 domains (nSH2, iSH2, or cSH2) were capable of forming liquid droplets independently (Fig. 3h). However, when combinations of two SH2 domains were tested, only co-presence of iSH2 and cSH2 resulted in droplet formation (Fig. 3h). Consistently, icSH2 fused with EGFP also formed liquid droplets (Fig. 3i). Notably, as low as 50 nM p85β could form liquid droplets, and p85β condensates were disrupted by high concentration of NaCl (Fig. 3g and 3i).

To further investigate the functional relevance of LLPS, we generated a p85β mutant lacking the cSH2 domain (p85β-Δc) (Fig. 3e). Unlike wild-type p85β, p85β-Δc diffused uniformly throughout the nucleus without forming visible droplets in HCC cells (Figure 3k). Moreover, overexpression of p85β-Δc failed to restore colony formation defects caused by p85β knockdown (Fig. 3j). Moreover, overexpression of p85β-Δc failed to restore the growth defects caused by p85β knockdown of xenograft tumors (Fig. 3l, 3m, 3n and Fig. S3c). These results demonstrate the critical role of p85β phase separation in the nucleus for its oncogenic function in HCC tumorigenesis.

### p85β colocalizes with POLR1A in the nucleoli of HCC cells

**Figure 4:**
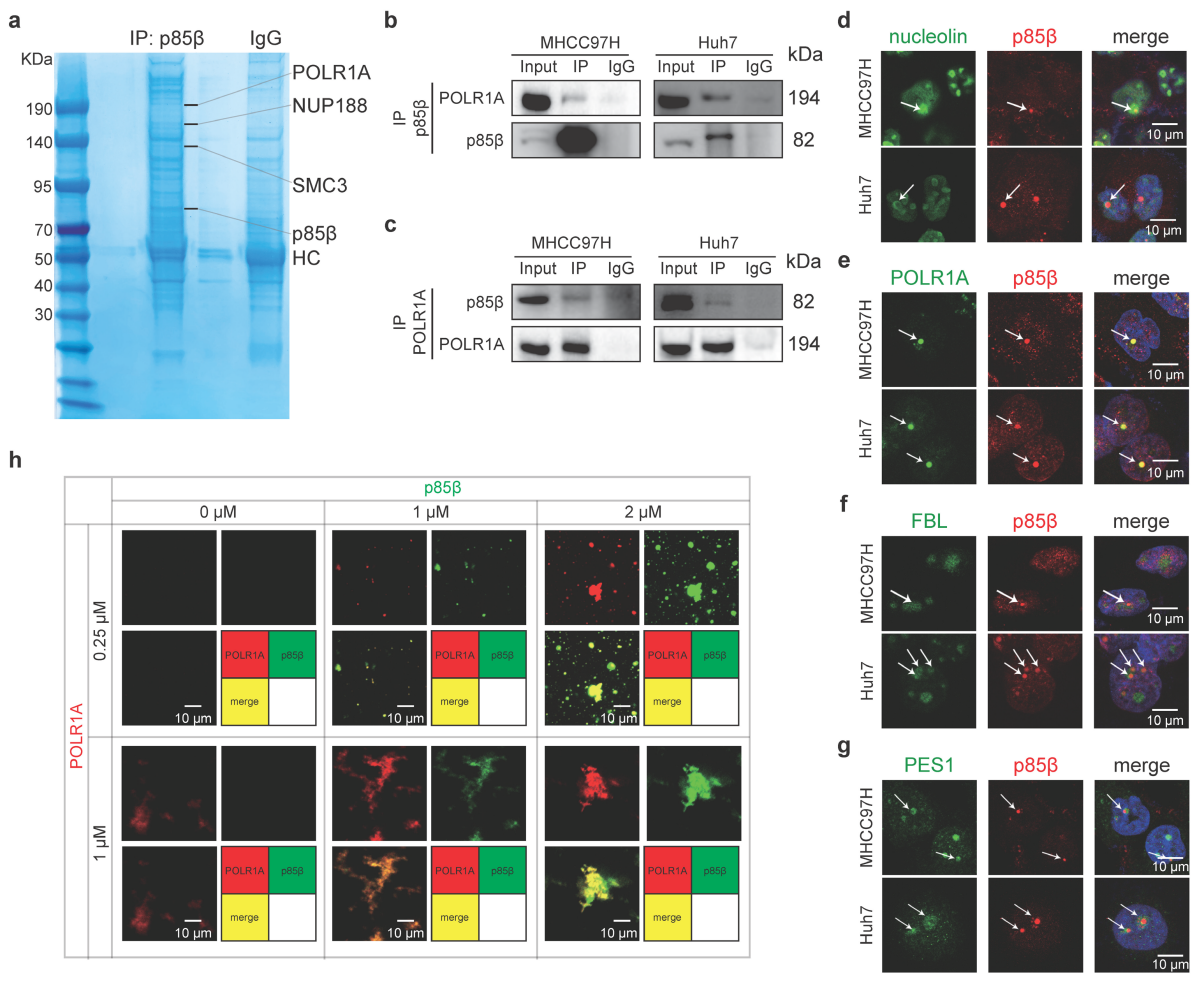
** p85β colocalizes with POLR1A in fibrillar center of the nucleolus. a-c** p85β interacts with RNA Pol I subunit POLR1A. Identifying the interaction proteins of p85β by mass spectrometry in nuclear lysates of 293T cells (**a**). The interaction between p85β and POLR1A was validated with co-immunoprecipitation by p85β antibodies **(b)** or POLR1A antibodies **(c)** in nuclear lysates of HCC cells. **d-g** p85β colocalizes with POLR1A in fibrillar center of the nucleolus. Co-immunostaining of p85β with nucleolus marker nucleolin **(d)**, fibrillar center marker POLR1A **(e)**, dense fibrillar component marker FBL **(f)**, or granular component marker PES1** (g)**. **h** p85β colocalizes with POLR1A *in vitro.* p85β-EGFP and POLR1A-AF555 proteins were purified* in vitro*. Co-localization and co-phase separation of p85β and POLR1A were analyzed.

To explore the composition of nuclear p85β condensates, we isolated nuclei from HEK293T cells and identified potential p85β-interacting proteins. Several candidates including nucleolar RNA polymerase I subunit POLR1A, nuclear pore complex protein NUP188, and chromosomal cohesion factor SMC3 were identified by mass spectrometry (Fig. 4a). Gene Set Enrichment Analysis (GSEA) of LIHC TCGA data also demonstrated a significant correlation between PIK3R2/p85β expression and pathways involved in rRNA processing and ribosome assembly (Fig. S4b), implying a potential role for p85β in regulating rRNA synthesis via POLR1A, the catalytic core subunit of RNA polymerase I. The interaction between POLR1A and p85β was further confirmed by co-immunoprecipitation in HCC cells nuclear extracts (Fig. 4b and 4c).

The nucleolus, the largest membraneless organelle in eukaryotic nuclei, is designed to fulfill the need for large-scale production of rRNAs and assembly of the ribosomal subunits 19. It comprises three distinct subcompartments including fibrillar center (FC), dense fibrillar component (DFC) and granular component (GC), which are complicated but well-organized by phase separation 19. As expected, p85β condensates were observed within the nucleolus, as indicated by colocalization with the nucleolar marker nucleolin (Fig. 4d). Specifically, p85β condensates were localized in fibrillar center of nucleolus, as evidenced by their precise overlap with POLR1A (an FC marker), while being encircled by DFC marker FBL and GC marker PES1(Fig. 4e-4g). *In vitro* experiments also demonstrated that recombinant p85β-EGFP and POLR1A-AF555 proteins exhibited nearly complete co-localization (Fig. 4h). Notably, POLR1A itself cannot undergo LLPS independently *in vitro*, but the presence of p85β promoted the formation of POLR1A condensates (Fig. 4h). Interestingly, high p85β/POLR1A ratio gradually facilitated the aggregation of POLR1A into compact FC-like structures (Fig. 4h), suggesting that overexpression of p85β in HCC may have the potential to maintain nucleolar structure. Furthermore, the colocalization of POLR1A and p85β were also detected in other cancer cell lines (Fig. S4c), suggesting a general role of p85β in nucleolar function across various cancer types.

Together, these findings suggest that p85β phase separation may critically regulate nucleolar organization and rRNA processing through its interaction with POLR1A.

### p85β stabilizes POLR1A by USP7

**Figure 5:**
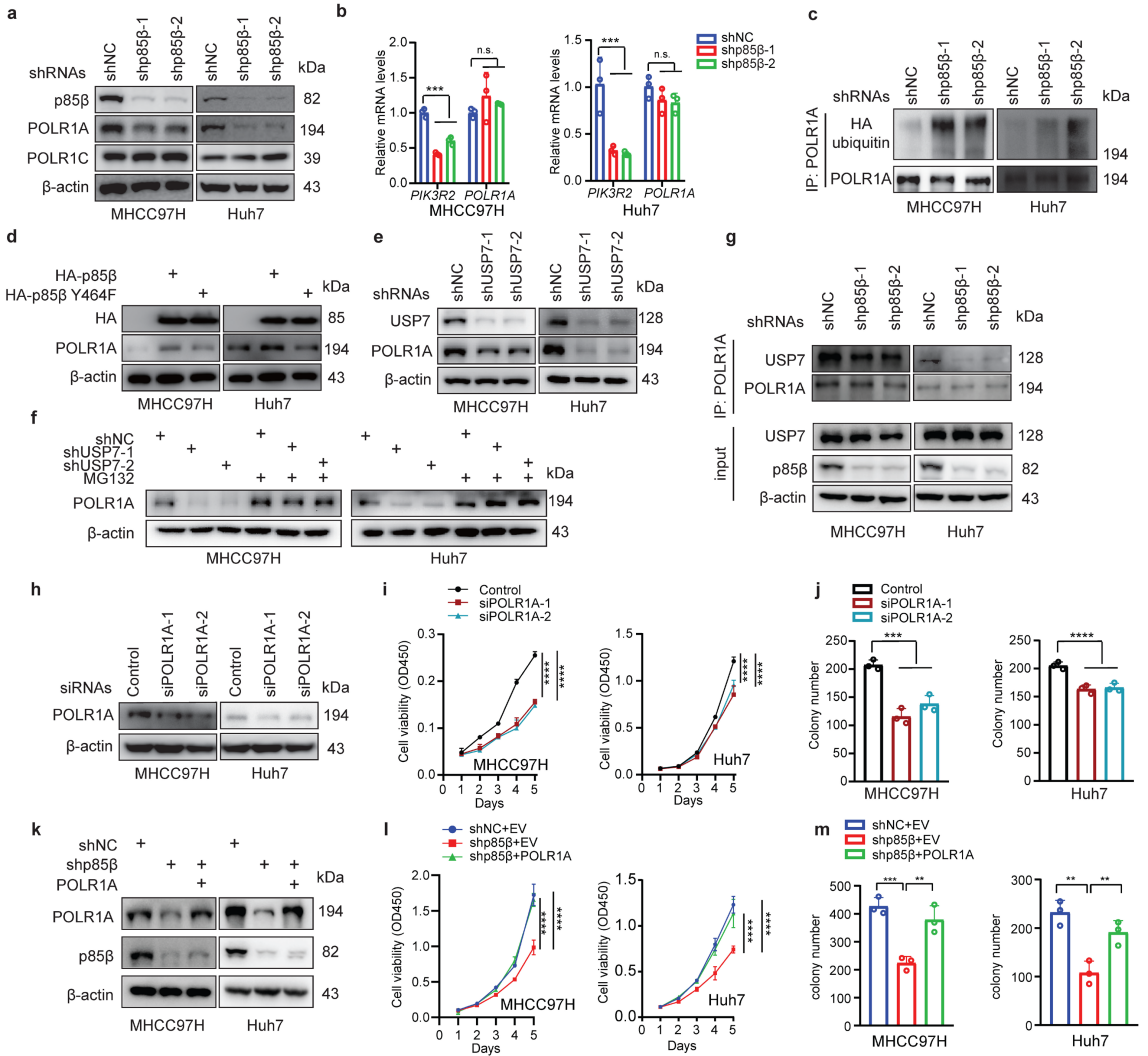
** p85β impacts HCC growth through stabilizing POLR1A a-c** p85β stabilizes POLR1A. p85β knockdown reduced the protein levels of POLR1A (**a**) without altering POLR1A mRNA levels (**b**). Knockdown of p85β increased ubiquitination of POLR1A (**c**). **d** Wild-type p85β but not nucleus-excluded p85β (p85β Y464F) restored POLR1A protein levels. **e** Knockdown of USP7 reduced POLR1A protein levels. **f** Proteasome inhibitor MG132 restored POLR1A protein levels in USP7 knockdown cells. **g** Immunoprecipitation analyses showed that knockdown of p85β reduced the binding of USP7 to POLR1A in HCC cells.** h-j** Knockdown of POLR1A inhibits HCC cell proliferation. Indicated cell lines were assayed for POLR1A knockdown efficiency (**h**), proliferation rates (n=5) (**g**) and colony formation (n=3) (**j**). **k-m** POLR1A rescued the proliferation (n=5) (**l**) and colony formation ability (n=3) (**m**) of p85β knockdown HCC cells. n.s. not significant, **p < 0.01, ***p < 0.001, ****p < 0.0001.

p85β prevents its binding proteins from ubiquitination-mediated degradation in the nucleus by recruiting the deubiquitinase USP7^11^, ^12^. Base on this mechanism, we hypothesized that p85β may regulate POLR1A stability. As expected, knockdown of p85β reduced the protein levels of POLR1A specifically, but not another subunit of RNA Pol I POLR1C (Fig. 5a). Knockdown of p85β had no impact on POLR1A mRNA levels (Fig. 5b), but significantly increased the ubiquitination of POLR1A proteins (Fig. 5c). POLR1A protein levels were largely rescued by overexpression of wild-type p85β but not nucleus-excluded p85β Y464F mutant (Fig. 5d), suggesting that p85β regulates POLR1A stability in the nucleus. p85β stabilized POLR1A by recruiting USP7 because knockdown of USP7 significantly reduced POLR1A protein levels (Fig. 5e) and proteasome inhibitor MG132 treatment largely rescued POLR1A protein levels which were reduced by USP7 knockdown (Fig. 5f). Immunoprecipitation analyses showed that knockdown of p85β reduced the binding of USP7 to POLR1A in HCC cells (Fig. 5g). Taken together, these data suggest that p85β recruits USP7 to stabilize POLR1A.

### p85β impacts HCC growth through POLR1A

We next determined whether p85β regulated HCC growth through POLR1A. Similar as knockdown of p85β, knockdown of POLR1A also reduced the proliferation and colony formation of HCC cells (Fig. 5h-5j). Overexpression of POLR1A restored the proliferation and colony formation of p85β knockdown cells (Fig. 5k-5m). These data suggest that p85β regulated HCC tumorigenesis through POLR1A.

### p85β regulates rRNA transcription to modulate the stemness of HCC cells

**Figure 6:**
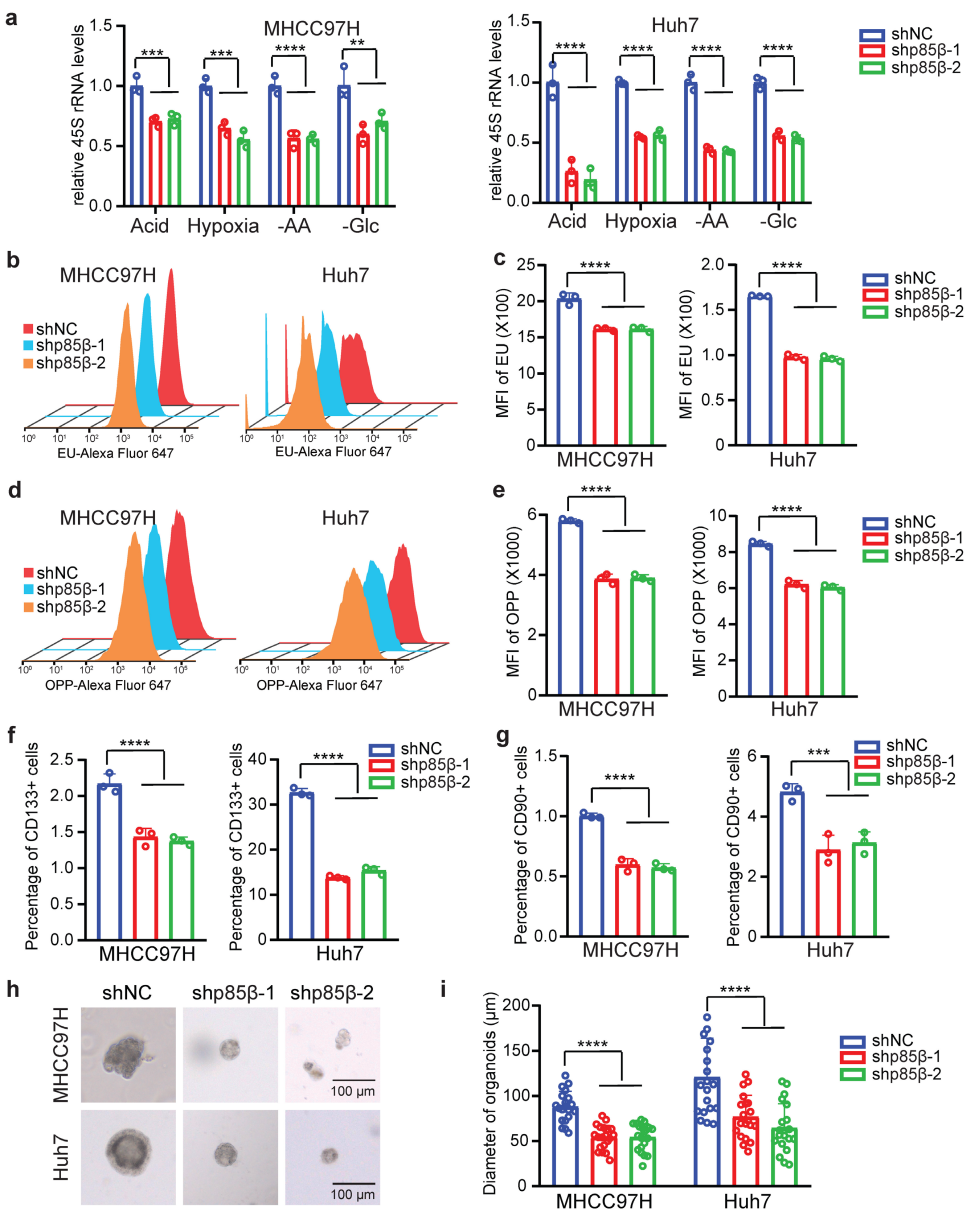
** p85β regulates rRNA transcription to modulate the stemness of HCC cells. a-e** Knockdown of p85β impaired rRNA synthesis. Knockdown of p85β decreased 45S pre-rRNA levels of HCC cells under tumor microenvironment conditions (n=3) **(a)**. Knockdown of p85β impaired total RNA synthesis by EU assay (n=3) **(b, c)** and total protein synthesis by OPP assay (n=3)** (d, e)**. **f-i** Knockdown of p85β impaired the stemness of HCC cells (n=3). Percentage of CD133+ cells **(f)** or CD90+ cells **(g)** was decreased by p85β knockdown. The diameter of organoids formed by HCC cells were reduced by p85β knockdown (n=20) **(h, i)**. n.s. not significant, **p < 0.01, ***p < 0.001, ****p < 0.0001.

Given the role of p85β in stabilizing POLR1A, we hypothesized that p85β regulates RNA Pol I activity. Indeed, knockdown of p85β significantly reduced pre rRNA (45S rRNA) transcription under various tumor microenvironments such as acidity, hypoxia, amino acid limitation or glucose limitation (Fig. 6a). Moreover, knockdown of p85β significantly reduced the global RNA transcription (rRNA accounts for 80%) (Fig. 6b and 6c), which could potentially impair the ribosome assembly, thereby resulting in a subsequent decline in global protein synthesis (Fig. 6d and 6e).

Notably, high levels of POLR1A have been characterized as the feature of stem cell hierarchy for colorectal cancer development 20. We therefore investigated whether p85β regulates HCC stemness via POLR1A. CD133 28 and CD90 29 have been identified as markers of tumor-initiating HCC cells. p85β knockdown significantly reduced the proportion of CD133+ or CD90+ HCC cells (Fig. 6f, 6g, S5a and S5b). Moreover, p85β depletion significantly decreased tumorosphere size in 3D culture systems (Fig. 6h and 6i). These data suggest that p85β enhances HCC stemness and tumorigenesis through POLR1A.

### p110α-ABD peptides inhibit HCC growth by targeting p85β condensates

**Figure 7:**
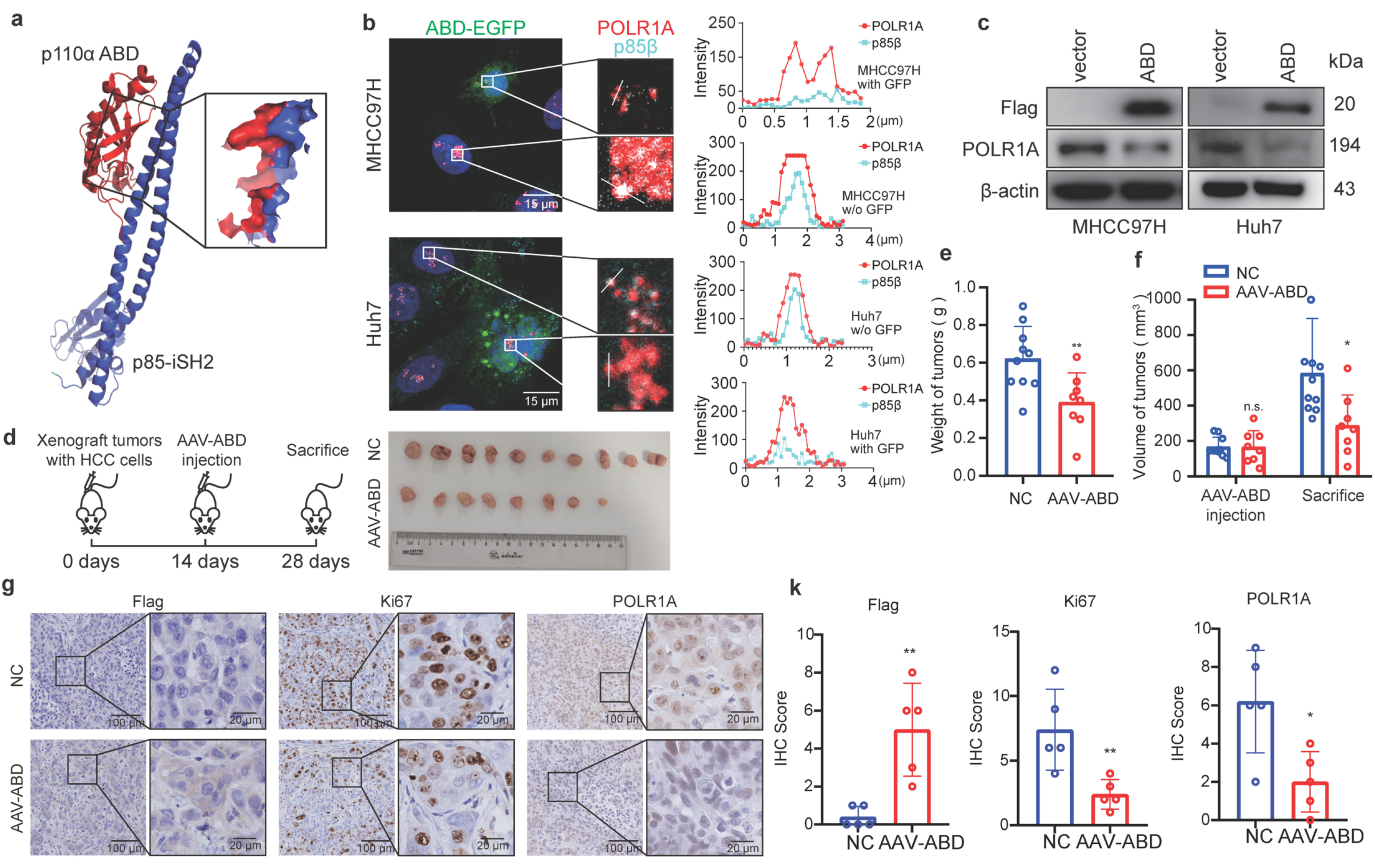
** p110α-ABD domain inhibits HCC growth by targeting p85β condensates. a, b** Expressing a peptide containing p110α ABD domain blocks colocalization of p85β and POLR1A. The protein structure of interface between p110α ABD domain and p85β iSH2 domain (structure data from Protein Data Bank, ID: 7PG5) **(a)**. The colocalization of nuclear p85β and POLR1A were impaired by overexpression of ABD-EGFP **(b)**. **c-g** p110α**-**ABD inhibits HCC tumor growth. Overexpression of p110α ABD domain reduced POLR1A protein levels in HCC cells** (c)**. AAV-ABD treatment inhibited the growth of xenograft tumors by MHCC97H cells** (d)**. Final tumor weights **(e)** and tumor volumes before and after AAV-ABD treatment **(f)** were presented (NC=10, AAV-ABD=8). AAV-ABD treatment reduced Ki67 positive cells and POLR1A expression in xenograft tumors** (g)**. Quantification of IHC staining of Flag, Ki67 and POLR1A (n=5)** (k)**. *p < 0.05, **p < 0.01.

Given the critical role of p85β condensates in HCC progression, its blockade emerges as a promising therapeutic strategy. Both p85β and p85α tightly bind the ABD domain of p110α in the cytoplasm 30 (Fig. 7a), thus, p110α-ABD peptide may disrupt p85β/POLR1A colocalization to destabilize POLR1A by competitively binding to p85β. We then engineered a FLAG-ABD-EGFP construct and overexpressed it into cells. Consistent with our hypothesis, p85β and POLR1A colocalization were disrupted in cells expressing FLAG-ABD-EGFP, but not in cells lacking EGFP signal (Fig. 7b). Consequently, FLAG-ABD overexpression resulted in a pronounced downregulation of POLR1A expression (Fig. 7c), recapturing the effects observed with p85β knockdown or blocking p85β nuclear translocation (Fig. 5a and 5d). Moreover, AAV-mediated delivery of FLAG-ABD markedly suppressed the growth of MHCC97H-derived xenograft tumors (Fig. 7d-7f), and reduced Ki67+ cells and POLR1A expression in these tumors (Fig. 7g and 7k). These findings suggest that p110α-ABD peptides can target p85β/POLR1A condensates to inhibit HCC growth.

### Delivering p110α-ABD peptide by circRNA simultaneously targets p85β/POLR1A condensates and suppresses PI3K/AKT signaling in HCC

**Figure 8:**
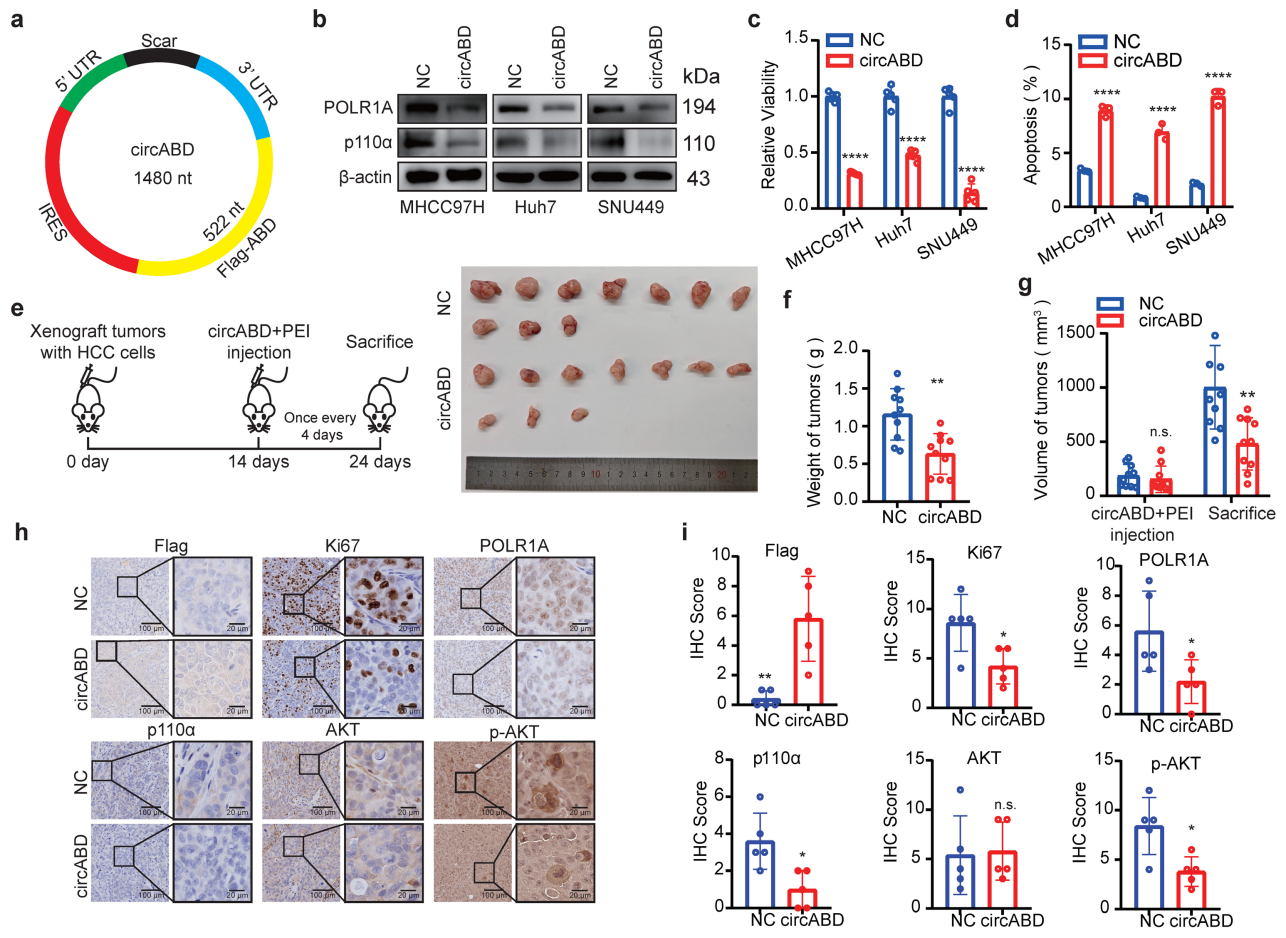
** Delivering p110α-ABD peptide by circRNA simultaneously targets p85β/POLR1A condensates and suppresses PI3K/AKT signaling in HCC. a** The schematic of an engineered circRNA expressing ABD peptide (circABD). **b** circABD treatment decreased the expression of POLR1A, p110α and phosphorylation of AKT. **c** circABD treatment reduced the viability of HCC cells (n=5). **d** circABD treatment induced the apoptosis of HCC cells (n=3). **e-g** circABD treatment inhibited the growth of xenograft tumors (n=10). Mice harboring subcutaneous xenograft tumors generated by MHCC97H cells were treated with vehicle or circABD. Representative tumor pictures **(e)** and final tumor weights **(f)** were presented. Tumor volume was measured before and after circABD treatment **(g). h** IHC staining of Flag-ABD, Ki67, POLR1A, AKT and AKT S473 were analyzed. Quantification of IHC staining of Flag-ABD, Ki67, POLR1A, AKT and AKT S473 (n=5)** (i)**. **p < 0.01, ****p < 0.0001.

To advance towards clinical application, we explored various ABD peptide delivery strategies and ultimately selected circular RNA expressing FLAG-ABD (circABD) due to its superior stability and expression efficiency (Fig. 8a, S6a and S6b). The *in vitro* synthesized circABD showed robust intracellular expression of FLAG-ABD peptides, achieving levels comparable to those of FLAG-ABD constructs (Fig. S6c), which subsequently resulted in a significant downregulation of POLR1A expression (Fig. 8b). Interestingly, circABD treatment also effectively decreased the expression of p110α and phosphorylation of AKT (Fig. 8b), as the ABD peptide could also compete with p110α for binding to p85α and p85β, ultimately leading to p110α degradation. These findings indicate that circABD possesses dual inhibitory functions, targeting both p85β/POLR1A condensates and PI3K/AKT signaling pathways.

Subsequently, we assessed the therapeutic potential of circABD for HCC treatment. As expected, circABD treatment markedly reduced the viability and induced the apoptosis of HCC cells (Fig. 8c and 8d). RNA-seq analysis further confirmed that circABD treatment induced apoptosis in HCC cells (Fig. S6d and S6e). Moreover, circABD treatment inhibited the growth of xenograft tumors generated by MHCC97H (Fig. 8e-8g). Consistent with our hypothesis, circABD treatment not only reduced the expression of POLR1A, but also decreased the expression of p110α and phosphorylation of AKT in xenograft tumors (Fig. 8h and 8i).

## Discussion

In this study, we demonstrate the function and molecular mechanism of p85β in HCC development. Although the oncogenic role of p85β has been recognized, the precise mechanisms underlying its tumorigenic functions remain controversial. Previous studies have demonstrated that cytoplasmic p85β promotes tumorigenesis by regulating PI3K activity in lung, colon, and breast cancer 25-27. However, the abundance of regulatory subunits p85α and p85β exceed those of catalytic subunits p110α and p110β leading to the existence of p110-free p85s in normal tissues 13. Notably, p85β is further overexpressed and correlates with poor prognosis across multiple cancer types, implying that free p85β might participate in human cancers independent of PI3K regulation. Our recent findings have revealed that nuclear p85β drives tumorigenesis through regulating histone methylation in PIK3CA helical domain mutant tumors and ccRCC 11, 12. In this article we found knockdown of p85β in HCC cells did not reduce the protein levels of EZH1 and EZH2. Similarly, in our previous article, we found that knocked down EZH1 or EZH2 in HCT116 (colon cancer) or Huh-7 cells (liver cancer), the expression of RB1, as well as its target genes (CCNA2, CCNE1 and CDK1), was not altered 11, 12, 25-27. Those results showed the mechanism of nuclear p85β regulating tumors may exhibit tissue specificity. We now provide compelling evidence that p110-free p85β exerts oncogenic functions in the nucleus of HCC cells. These findings highlight p110-free p85β as an emerging therapeutic target in human cancer.

Although p85 proteins have long been recognized as PI3K regulatory subunits, emerging evidence suggests their additional roles as adaptor proteins in various complexes independent of PI3K. For instance, p85α nSH2 mutant proteins enhance HER3 (ERBB3) interaction, stabilizing HER3 to activate EGFR, HER2, and c-MET signaling for tumorigenesis 31. Similarly, p85β stabilizes receptor tyrosine kinase AXL to induce oncogenic signaling in ovarian cancer 32. Beyond receptor tyrosine kinase regulation, p85β depletion reduces BRD4 protein levels, impairing DNA damage repair 33. Moreover, p85β and p85α can stabilize BRD7 proteins for glucose homeostasis 34, and nuclear p85β can stabilize EZH1 and EZH2 for cancer progression 11, 12. Given the well-established role of p85 proteins in stabilizing p110 subunits, it is reasonable to hypothesize that p85 proteins function as molecular adaptors to regulate the stability of their interacting partners. In the current study, we have identified a novel function of p85β as an adaptor protein for stabilizing RNA polymerase I subunit POLR1A, regulating rRNA synthesis during HCC progression. Thus, exploring p85-mediated protein degradation system may advance our understanding of p110-free p85s.

We have uncovered a novel mechanism by which p85β modulates nucleolar function. Firstly, nuclear p85β prefers to localize in the fibrillar center of nucleoli in HCC cells. Secondly, p85β interacts with and stabilizes POLR1A, a crucial component of the nucleolar fibrillar center. Thirdly, p85β knockdown significantly impairs rRNA transcription and global protein synthesis. Considering the strong binding affinity between p85s and p110s, coupled with widely presence of nuclear-localized p85 proteins, it is conceivable that p110s may also exist in the nucleus, potentially even in the nucleolus to regulate biological processes. Indeed, Kumar *et al*. reported p110β located in the nucleus of MEF cells 35, while Mazloumi Gavgani *et al*. demonstrated nucleolar localization of both p110β and its product PIP3 in endometrial cancer cells 36. Consequently, AKT can be activated in the nucleus 36, 37. Whether PI3Kβ/AKT have function in nucleoli to facilitate cancer progression worth investigating in the future.

Our study reveals the critical role of p85β phase separation in HCC progression. Although p85s puncta have been previously observed in various contexts, for instance p85β in focal adhesions of 3T3 cells 38, p85α in IRS1-driven liquid droplets in insulin signaling 39, and p85α/p110α puncta at endosomes for AKT activation 40, 41, none have implicated p85 proteins as drivers of membraneless organelles through LLPS. In this study, we demonstrate that p85β can undergo LLPS intramolecularly by itself and intermolecularly with POLR1A. Despite the absence of classic intrinsically disordered regions (IDRs) predicted by IDR analysis software, we identify that iSH2 and cSH2 domains are crucial for p85β LLPS. High concentration of NaCl could disrupt p85β LLPS, suggesting that electrostatic interactions may be critical for p85β LLPS. The conservation of iSH2 and cSH2 domains in both p85α and p85β implies potential LLPS capability in p85α as well.

Given the tumorigenic role of p85β LLPS, targeting p85β condensates represents a promising therapeutic strategy for HCC treatment. While peptides have been considered as a strategy to disrupt LLPS structure 42, how to deliver peptide into the cells feasibly and economically still remains challenging. Messenger RNA (mRNA)-based vaccines show profound applications in infectious diseases and cancer especially after COVID-19 pandemic because they are much safer without infection or integrating and can be rapidly and inexpensively synthesized with high yield through *in vitro* transcription 43. After evaluating various delivery strategies, we selected circular RNA (circRNA) due to its feasibility, low cost, and minimal immunogenicity compared to linear mRNA counterparts, thereby expanding its therapeutic potential in oncology 44, 45. Our study reveals a peptide containing p110α ABD domain, which tightly binds p85s to target p85β/POLR1A condensates. This peptide disrupts the spatial colocalization of p85β and POLR1A, promotes POLR1A degradation, and consequently suppresses HCC tumorigenesis. We finally developed an optimized circRNA to express p110α ABD peptide. Interestingly, the engineered circABD not only disrupts p85β/POLR1A condensates but also inhibits PI3K/AKT signaling by promoting p110α degradation. This bifunctional therapeutic strategy with circABD, which capitalizes on the convergence of phase separation biology and oncogenic signaling pathways, ideally enhanced its therapeutic efficacy for HCC, and positions circABD as a paradigm-shifting approach in RNA-based cancer therapeutics.

## Methods

### Cell culture and transfection

All cell lines were maintained at 37°C in a humidified atmosphere with 5% CO_2_. HEK293T, MCF-7, SK-MES-1, Capan-1 and HCC cell lines (Huh7, MHCC97H, HepG2 and SNU449) were cultured in DMEM medium, and DLD-1, Hgc-27, TOV-21G were cultured in RPMI-1640 medium. Medium was supplemented with 1% of Pen/Strep (Gibco) and 10% of fetal bovine serum (Gibco). The cell lines were authenticated by the Genetica DNA Laboratories using STR profiling.

The siRNAs or shRNAs targeting human PIK3R2, POLR1A and the scramble control were all purchased from Biotend (Shanghai, China). The plasmids and siRNAs were transfected with *Trans*IT-X2^®^ Dynamic Delivery System (Mirusbio, Cat# MIR 6000) according to manufacturer's instruction. The circRNA were transfected with Trans®Polyethylenimine Linear (YEASEN, Cat# 40816) according to manufacturer's instruction. The sequences of siRNAs were listed in Table S1.

### Human HCC sample

Human HCC samples were collected from Department of Liver Surgery, Renji hospital, School of Medicine, Shanghai Jiao Tong University. The HCC were diagnosed by pathology. All procedures involving the collection and application of human samples were approved by the Ethics Committee of Renji Hospital and adhered to the principles of the Declaration of Helsinki. Written informed consent was obtained from human participants or their family members.

### Mouse models

The 6-8 weeks old female nude mice were utilized for subcutaneous xenograft tumor models. To monitor the xenograft tumor growth, two million cells were injected subcutaneously and bilaterally into both flanks of nude mice. Tumor weights and volumes were measured and analyzed as described previously 46. To examine the therapeutic effects of indicated AAV virus or circRNAs, five million cells were injected subcutaneously and bilaterally into both flanks of nude mice. Once xenograft tumors start to grow, mice were randomly divided into two groups for vehicle or treatment. Tumor volumes were measured before treatment and after sacrifice. Tumor weights were measured after sacrifice. Indicated AAV virus (ten billion VG/tumor, generated by Cyagen Biosciences) or circular RNAs coated with PEI (20 μg/tumor) were directly injected into xenograft tumors.

Two transgenic mouse strains, *Pik3r2 ^Y458F^* knock-in mice and *Pik3r2^em1flox^* conditional knock-out mice were generated with C57BL/6 background by Cyagen Biosciences. *Pi3kr2* was knocked out in liver by TBG-Cre AAV8 (ten billion VG/mouse, Cyagen Biosciences) through tail vein injection. Spontaneous hepatocellular carcinoma was induced as reported previously 47. In brief, 5 μg/mouse SB100, 7.5 μg/mouse myr-AKT1 and 7.5 μg/mouse N-RasV12 were diluted in 2 mL/mouse sterile saline (0.9 % NaCl) and injected through the tail vein in 5 to 7 seconds.

### Cell growth assay

For CCK-8 (DOJINDO, Cat#CK04) assay, the cells were seeded into 96 well plate with 1000 cells/well. At indicated time points, CCK-8 reagent was diluted with full medium at ratio of 1:10 and the cells were cultured within this medium mixture for 90 min at 37°C. Then absorbance of OD450 was measured. Relative cell viability was calculated by treatment OD450/vehicle OD450 × 100%.

For colony formation assay, the cells were seeded into 6 well plate with 1500 cells/well and cultured for 2 weeks. Then cells were stained by Crystal Violet Solution and colonies were counted.

### Apoptosis assay

Cells were trypsinized after treated by circABD for indicated time, and then were stained by BD Pharmingen™ FITC Annexin V Apoptosis Detection Kit I (BD, Cat# 556547) according to manufacturer's instruction. Apoptosis cells were detected by flow cytometry.

### Real time PCR

RNA was extracted and purified with RNA extraction kit (fastagen, Cat#220011). cDNA was produced by PrimeScript™ II 1st Strand cDNA Synthesis Kit (Takara, Cat#6210) with Oligo dT Primer (mRNA) or random 6 mers (rRNA). The gene expression levels were measured by AceQ qPCR SYBR Green Master Mix (Vazyme, Cat#Q111) and analyzed by 2^-ΔΔct^. The sequences of primers were listed in Table S1.

### Nuclear protein extraction and Immunoprecipitation

Nuclear protein was extracted with lysis buffer containing 0.1% NP40 in PBS solution supplemented with complete Protease Inhibitor and PhosSTOP. The cell pellets were resuspended by lysis buffer and pipetted up and down several times with 1 mL peptide tips. Lysates were centrifuged at 12000 rpm for 1 min immediately. Pellets were then lysed in immunoprecipitation buffers (50 mM Tris-HCl at pH 7.5, 1mM EDTA at pH 8.0, 150mM NaCl, 1% NP-40, cOmplete Protease Inhibitor, PhosSTOP, and PMSF) by ultrasonic as nuclear lysates. Then immunoprecipitation was performed by indicated primary antibody and protein A/G beads. Lastly, the results were analyzed by Western blotting. The information of antibodies was listed in Table S1.

### Global RNA transcription and protein synthesis

The synthesis rate of global RNAs and proteins were detected by Click-iT® RNA Imaging Kits (ThermoFisher, Cat#C10330) and Click-iT® Plus OPP Protein Synthesis Assay Kits (ThermoFisher, Cat# C10458) respectively according to manufacturer's instruction. Briefly, the cells were cultured with full DMEM medium containing 1mM EU for 5 mins to detect the synthesis rate of rRNA. The cells were cultured with full DMEM medium containing 20μM OPP for 30 mins to detect the synthesis rate of protein. Then the cells were fixed, permeabilized and Click-iT® reacted with Alexa Fluor® 647 picolyl azide according to manual. The cells were detected by flow cytometry.

### Recombinant protein purification

The wild-type or truncated p85β fused with fluorescent proteins were cloned into pET-28a and recombinant proteins were expressed by BL21 *Escherichia coli* as described previously 48. When OD_600_ of bacterial fluid reached 0.4 ~ 0.6, 25 mg/L IPTG was added to fluid and the bacterial cells were cultured in 16°C with 150 rpm overnight. Then the bacterial cells were lysed by ultrasonic and the recombinant proteins were purified with Ni-NTA agarose according to HIS-tagged protein purification handbook from Qiagen. Finally, the elution buffer containing recombinant proteins were dialyzed with buffers including 100 mM NaCl, 50 mM Tris-HCl, pH = 7.5, 10% glycerol, and 1 mM DTT.

The ORFs of human POLR1A was sub-cloned into modified pCAG vector. The plasmid was then transfected to suspension Expi293F cells with PEI. After culture at 37°C, 5% CO_2_ for 3 days, cells were collected and lysed in the buffer containing 30 mM HEPES (pH 8.0), 300 mM NaCl, 0.25% CHAPS, 5 mM ATP, 5 mM MgCl_2_, 10% glycerol and 2 mM DTT at 4°C for 30 min, and the insoluble fraction was removed by centrifugation at 38,000 × g for 30 min. Supernatants were incubated with IgG antibody-agarose for 4 h and washed extensively. The fusion proteins were digested by ULP1 protease and then eluted with 30 mM HEPES (pH = 8.0), 300 mM NaCl, 0.1 % CHAPS, 2 mM MgCl_2_, 10% glycerol, 2 mM DTT. The protein was incubated with NHS-AF555 and dialyzed in buffer containing 50 mM HEPES 7.5, 100 mM NaCl, 5 mM DTT, and 10% glycerol.

### *In vitro* LLP

Purified proteins were diluted to various concentrations in LLPS buffer (50 mM Tris-HCl, pH=7.4, 100 mM NaCl, 1 mM DTT and 10% Glycerol). For each condition, 5 μl of protein solution was placed onto a glass slide, covered with a coverslip, and imaged using a confocal microscope (Zeiss). For LLPS assays, proteins were mixed in LLPS buffer containing indicated concentrations of NaCl, with or without PEG 8000, as specified in the figure legends.

### FRAP

FRAP experiments were performed in living Huh7 cells using a Leica SP8 confocal microscope equipped with a 63× oil immersion objective. Fluorescent labeled protein condenses were formed in Huh7 cells by expressing p85β-EGFP fused proteins. A circular region of 1 μm in diameter at the center of a condensate was bleached using 100% of the maximum power of the 405 nm laser. Fluorescence recovery at the bleached site was monitored over time. Recovery curves were analyzed using GraphPad Prism software.

### Circular RNA synthesis

The circular RNA was synthesized as recent report 45. The component of T4 thymidylate synthase (td) intron was used to circularize RNA. The sequences of IRES (iEV-B107) and 5'UTR (Apt-eIF4G) were chosen as described by Chen *et al*. 45. These sequences were cloned into plasmid containing T7 promotor, which were used as template for *in vitro* transcription (IVT) after linearization by restriction enzyme. RNA was synthesized with T7 High Yield RNA Transcription Kit 2.0 (Novoprotein, Cat#E332) at 37°C overnight by thermocycler according to manufacturer's instruction. The circular RNA was purified with RNase R (37°C, 1 h) and Lithium Chloride (7.5 M).

### Stem cell assays

The stem cell marker CD133^28^ or CD90^29^ was stained with antibody conjugated fluorescein in PBS after cells were digested with 2 mM EDTA. CD133 or CD90 positive cells were detected by flow cytometry.

For organoid assay, cells were seeded into Matrigel (Corning, Cat#356231) and cultured with Advanced DMEM/F-12 (Gibco, Cat#12634010) with 2 mM glutamine (Sigma, Cat#G3126), 10 mM Nicotinamide (Sigma, Cat#N0636), 1 mM NAC (Sigma, Cat#A9165), 10 μM Y-27632 (Abmole, Cat#M1817), 5 μM A83-01 (YEASEN, Cat#53002ES03), 1× B27 (YEASEN, Cat#60703ES10), 1× N2 (YEASEN, Cat#60706ES08), 50 ng/mL EGF (YEASEN, Cat#92703ES60), 100 ng/mL Noggin (YEASEN, Cat#92262ES08), 100 ng/L RSPO1 (YEASEN, Cat#92277ES60),10 nM Gastrin (YEASEN, Cat#53007ES03), 1 mM HEPES and 1% Penicillin-Streptomycin for 2 weeks. Then the diameters of organoids were analyzed with microscope.

### RNA-seq

The RNA was isolated by Trizol from Huh7 cell treated with vehicle or circular RNA overnight. Total RNA was qualified and quantified as follows: (1) RNA purity and concentration were examined using NanoDrop 2000; (2) RNA integrity and quantity were measured using the Agilent 2100/4200 system. After library preparation and pooling of different samples, the samples were subjected for NGS platform by Berry Genomics Co., Ltd (http://www.berrygenomics.com/, Beijing, China). To avoid reads with artificial bias (i.e., low-quality paired reads, which primarily result from base-calling duplicates and adaptor contamination), we removed the following types of reads: (i) reads with 3nt unidentified nucleotides; (ii) reads aligned to the adaptor; (iii) reads with ≥20% bases having phred quality score ≤5. Then, map the clean reads to the silva database to remove the rRNA by bowtie2 software. All the downstream analyses were based on the clean data without rRNA. Paired-end clean reads were aligned to the reference genome using HISAT2 software. Featurecount was used to count the reads numbers of each gene 49. Gene set enrichment assay was run by GESA software with KEGG database 50, 51.

### Cell immunofluorescence

Cells were seeded into glass directly or glass bottom dish with appropriate density. After 24 h, cells were fixed by 4% Paraformaldehyde and permeabilized by 0.1% Triton X-100. After 10% BSA incubation, cells were incubated in primary antibody with recommended concentration overnight at 4°C. Cells were then washed by PBS and incubated with secondary antibody conjugated with fluorescein. Lastly, cells were stained with DAPI and observed by confocal microscope.

### Immunohistochemistry

The paraffin-embedding and IHC staining of tissue samples were accomplished majorly by Servicebio company (Shanghai, China). Briefly, the tissues were fixed immediately by 4% paraformaldehyde followed by paraffin-embedding. After preheating section in 60°C, section was de-waxed by xylene and ethanol, and then 3% H_2_O_2_ were used to inhibit peroxisome. 10% BSA were applied for blocking to prevent non-specific binding of antibodies. Then sections were incubated with primary antibodies which were diluted according to antibody protocols in 4°C overnight. Next day sections were washed by TBST and incubated with secondary antibody conjugated with HPR. Lastly, DAB and hematoxylin were used to stain for antigen or nuclei respectively.

### Statistics

Two-tailed unpaired Student's t tests or two-way ANOVA were used for two group comparisons. Spearman's correlation test was performed to analyze the correlation. Survival curves were constructed using the Kaplan-Meier method with log rank test. Most data are shown as the mean ± SD. Tumor growth was shown as the mean ± SEM. p < 0.05 was considered statistically significant.

## Supplementary Material

Supplementary figures and table.

## Data Availability

The RNA-sequencing data have been deposited in the Gene Expression Omnibus (GSE291263). The datasets that support the findings of this study are available from the corresponding author (Yujun Hao: yjhao@shsci.org) upon reasonable request.
