# Supplementary material for: Phase separation of p85β modulates hepatocellular carcinoma progression through POLR1A
Source: Int J Biol Sci. 2026 May 18;22(10):5459–74. doi: 10.7150/ijbs.130598 (PMC13215363; doi:10.7150/ijbs.130598)
Supplement: Supplementary file 1 — Supplementary figures and table. [file ijbsv22p5459s1.pdf]

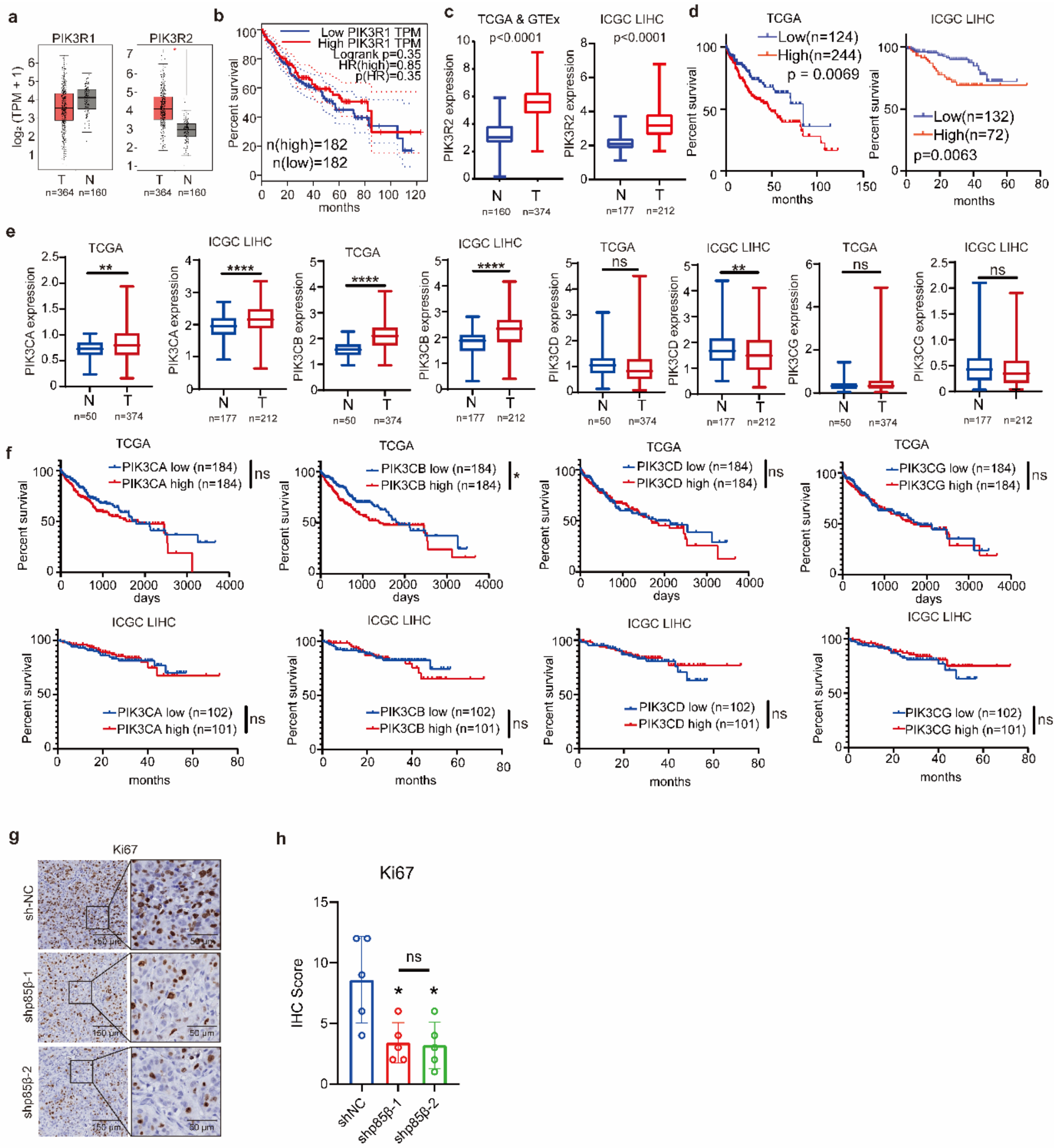

Fig. S1 p85β promotes HCC development. a The expression levels of PIK3R1/p85α and PIK3R2/p85β in HCC tumor samples (T) and normal tissues (N) were analyzed by GEPIA website. **b** PIK3R1/p85α is not correlated with overall survival of HCC patients (GEPIA). **c** PIK3R2/p85β is overexpressed in HCC tumor samples compared with normal tissues in TCGA and ICGC datasets. **d** High expression of p85β is correlated with worse overall survival of HCC patients in TCGA and ICGC datasets. **e** The expression levels of PI3K catalytic subunits in HCC using TCGA and ICGC datasets. **f** PI3K catalytic subunits is not correlated with overall survival of HCC patients. **g** IHC staining of Ki67 in xenograft tumors from control and p85β knockdown MHCC97H cells. Quantification of IHC staining of Ki67 (n=5). $*p < 0.05$, $**p < 0.01$.

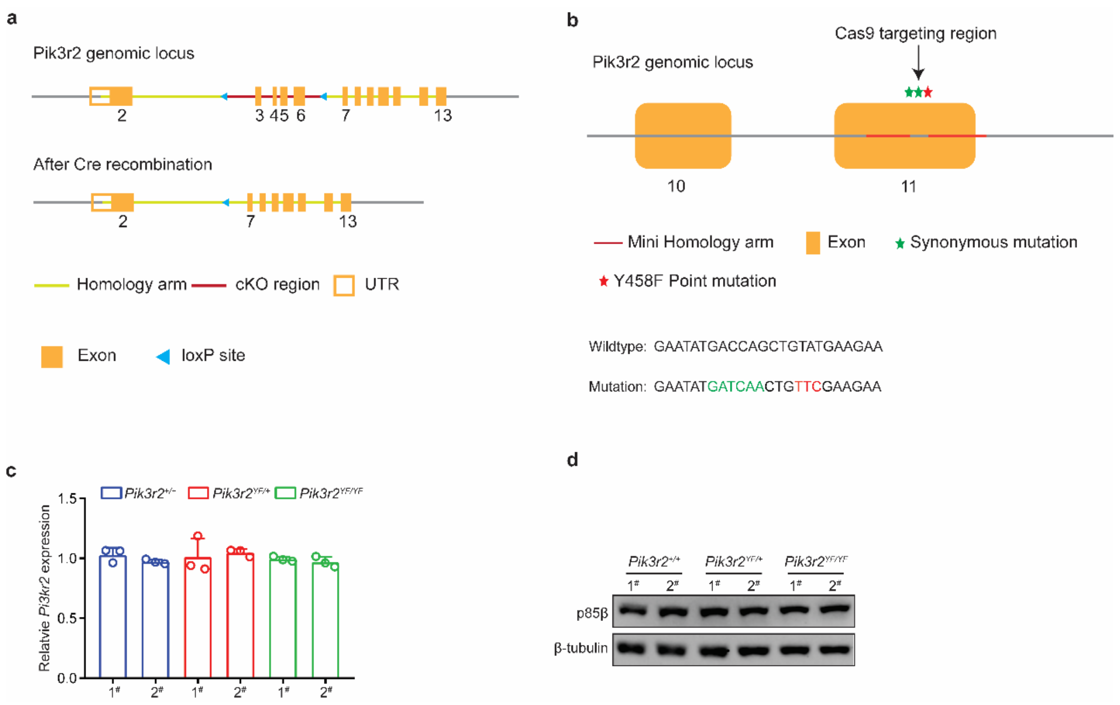

Fig. S2 A nucleus-exclusive p85β mutant mouse strain and Pik3r2 conditional knock out mouse strain were generated. **a** The schematic of *Pik3r2* conditional knock out mouse strain. Conditional Pik3r2 knockout C57BL/6J mice were generated by Cyagen Biosciences Inc (Suzhou, China) using Cre-loxP recombination system. Pik3r2 flox mice were generated by CRISPR-Cas9 targeted insertion of loxP recombination sites into introns flanking exon 3~6 of Pik3r2, excision of exon 3~6 (Pik3r2 Δ3~6) should cause severe carboxy-terminal deletion of the Pik3r2 protein and/or elimination of the mRNA by nonsense-mediated decay. The Cas9 protein, two sgRNA (sgRNA1: TTGAAGTGTAAAGCCCAGAG-TGG; sgRNA2:ATCCCTAAGTGAAAGGGAGC-AGG). And the targeting vector containing exon 3~6 flanked by two loxP sites and the two homology arms were co-injected into fertilized eggs. The embryos were transferred to recipient female mice to obtain F0 mice. The F0 founder mice were identified by PCR anaysis using two pairs of primers
(F1:5'-ACTCCAGTCTGGCAATTTTCTTTGA-3',
R1:5'-GCTTCTGTATTCAACTGGAACCG-3';
F2: 5'-CAGAAAGACTGGATCCCTAAGTGAA-3',
R2: 5'-AAGGAAAACAGGAAGTAATGCCAAG-3') and sequencing. F0 founder mice were bred to wild type mice to test germ line transmission and produce heterozygous F1. The genotype F1 mice was also confirmed by PCR. Pik3r2-flox mice were then cross-mated with XX-Cre mice to generate Pik3r2 fl/fl,XX-Cre mice. **b** The strategy of $Pik3r2^{Y458F}$ point mutation knock-in mouse strain. The mRNA levels **(c)** and protein levels **(d)** of Pik3r2/p85β were not impaired by $Pik3r2^{Y458F}$ mutation ($Pi3kr2^{+/+}$ =2, $Pi3kr2^{YF/+}$ =2, $Pi3kr2^{YF/YF}$ =2). The Pik3r2 p.Y458F point mutation C57BL/6J mice were generated by Cyagen Biosciences Inc (Suzhou, China). The mutations p.Y458F (TAT to TTC) in donor oligo were introduced into exon 11 of Pik3r2 gene (NM_008841.3) by homology-directed repair. Two silent mutations p.D455= (GAC to GAT) and p.Q456= (CAG to CAA) was also introduced upstream to prevent the binding

and re-cutting of the sequence after homology-directed repair. Cas9 protein, one gRNA (gRNA: GTGTGTATTCTTCATACAGCTGG) and a donor oligo were co-injected into fertilized eggs. The embryos were transferred to recipient female mice to obtain F0 mice. The F0 founder mice identified were by PCR using a pair of primers (F1:5'-CCTGTGTCCAAGTACCAACAAGT-3', R1:5'-AATCCCACTACAAACCCATCCTTG-3') and sequencing. F0 founder mice were crossed to C57BL/6J mice to test germ line transmission and produce heterozygous F1. The genotype F1 mice was also confirmed by PCR and sequencing.

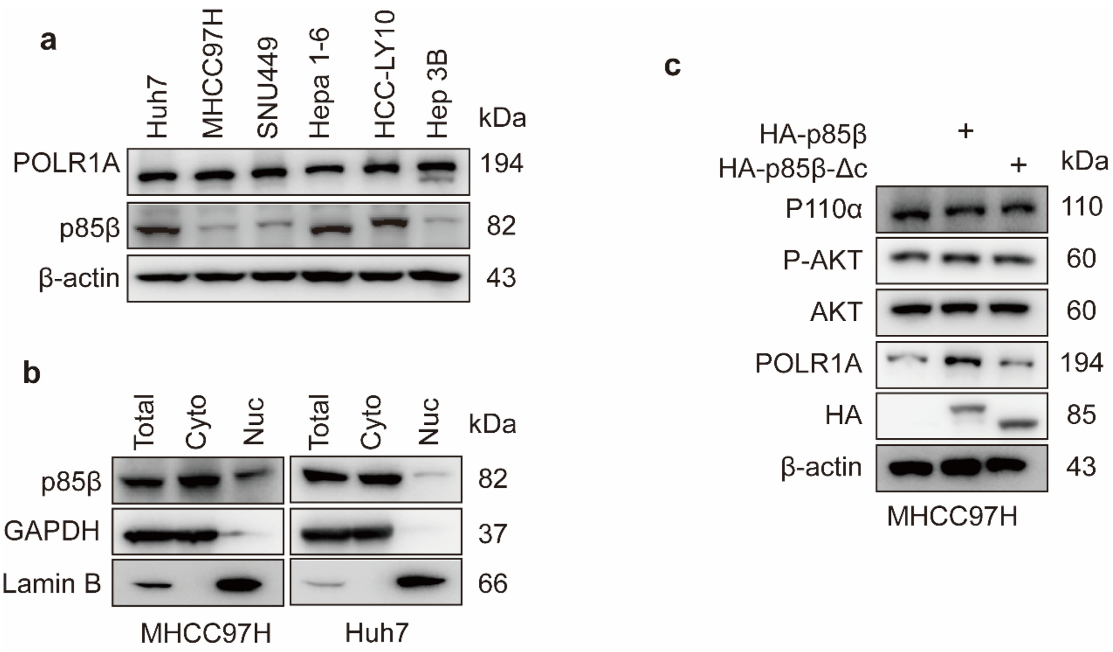

Fig.S3 Cyto/Nuc fractionation analyses of MHCC97H and Huh7 cells. a The endogenous expression levels of p85β and POLR1A in HCC cell lines (MHCC97H, Huh7, SNU449, Hepa 1-6, HCC-LY10 and Hep 3B). **b** Cytoplasmic (Cyto) and nuclear (Nuc) fractionation analyses of MHCC97H and Huh7 cells. **c** POLR1A protein levels were largely rescued by overexpression of wild-type p85β but not p85β-Δc.

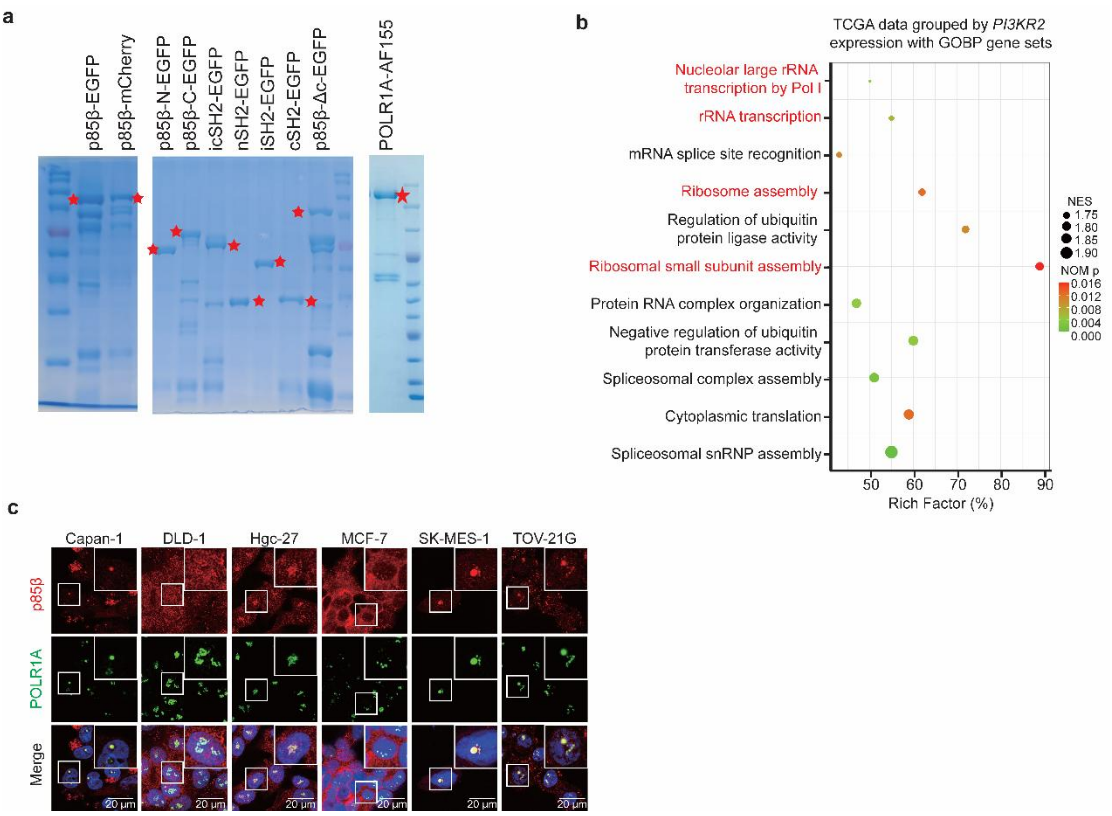

Fig.S4 p85β regulates rRNA synthesis through POLR1A. a Purified recombinant His-tagged p85β-EGFP, p85β-mCherry, p85β C-terminal-EGFP, p85β N-terminal-EGFP, individual SH2 domains (nSH2-EGFP, iSH2-EGFP, or cSH2-EGFP), icSH2-EGFP, p85β-Δc-EGFP and POLR1A-AF155 proteins were resolved on SDS-PAGE gels. Asterisk indicates purified recombinant proteins. **b** PIK3R2 expression levels correlate with rRNA synthesis and ribosome assembly in TCGA LIHC dataset. **c** p85β and POLR1A colocalized in the nucleus of various cancer cells by immunofluorescence.

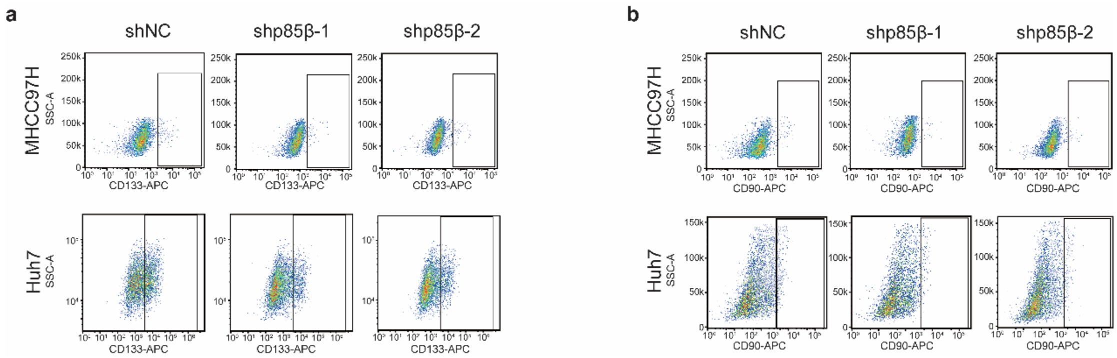

Fig. S5 p85β modulates stemness of HCC cells by POLR1A. Dot plots of CD133+ **(a)** or CD90+ **(b)** HCC cells.

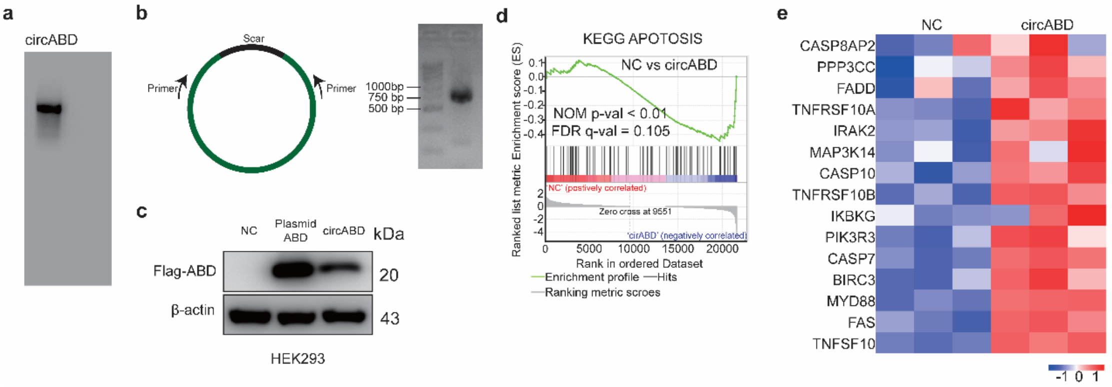

Fig. S6 A peptide encoding p110α ABD domain inhibited HCC tumor growth. a-c The synthesis and purification of an engineered circRNA expressing p110α ABD **(a)**. circABD was validated by RT-PCR **(b)**. circABD expressed comparable Flag-ABD as overexpression of ABD constructs **(c)**. **d, e** RNA-sequencing analysis illustrated that circABD treatment induced cell apoptosis.

Table S1 Antibodies and oligoes

Antibodies

**Name**	**Company**	**Cat#**
anti-FLAG	Sigma-Aldrich	F1804
anti-HA	Abways technology	AB0004
anti-p110α	Cell Signaling Technology	4249
anti-p85β	Abcam	ab180967
anti-p85β	abcam	ab131067
anti-p-AKT	Cell Signaling Technology	4060
anti-AKT	Cell Signaling Technology	9272
anti-p-GSK-3β	Cell Signaling Technology	9336
anti-GSK-3β	Cell Signaling Technology	9315
anti-p-mTOR	Cell Signaling Technology	5536
anti-mTOR	Cell Signaling Technology	2983
anti-p-ERK	Cell Signaling Technology	4370
anti-ERK	Cell Signaling Technology	4695
anti-H3K27me3	Cell Signaling Technology	9733
anti-EZH1	Cell Signaling Technology	62083
anti-EZH2	Cell Signaling Technology	62083
anti-POLR1A	santa cruz biotechnology	sc-48385
anti-POLR1C	proteintech	15923-1-AP
anti-USP7	proteintech	66514-1-Ig
ant-β-actin	proteintech	66009-1-Ig
anti-Ki67	Servicebio	GB111141
anti-Ki67	Servicebio	GB121499

Primers

**NAME**		**Sequence (5' to 3')**
β-actin	F	CATGTACGTTGCTATCCAGGC
	R	CTCCTTAATGTCACGCACGAT
POLR1A	F	GCGATCCGTTGTCCGAAAG
	R	GGTGGGTGTTAAGTATCCTCGTT
Pre-45s	F	GAAACCTTCCGACCCCTCTC
	R	TACGAGGTCGATTTGGCGAG
PI3KR2 (human)	F	AAAGGCGGGAACAATAAGCTG
	R	CAACGGAGCAGAAGGTGAGTG
Pik3r2 (mouse)	F	GGATGCCTGGCTTCAACGA
	R	CTGGGAGTATGTGGCCTGACT

siRNAs

NAME	Sequence (5' to 3')
si-POLR1A-1	UUCACCCGGGAGCACUAUAUGTT
si-POLR1A-2	UCUAGCACCAGCAAUACAUUUTT
